# Corrigendum: A single amino acid in coat protein of Pepper mild mottle virus determines its subcellular localization and the chlorosis symptom on leaves of pepper

**DOI:** 10.1099/jgv.0.002272

**Published:** 2026-05-13

**Authors:** Kelei Han, Hongying Zheng, Mengfei Ji, Weijun Cui, Shuzhen Hu, Jiejun Peng, Jinping Zhao, Yuwen Lu, Lin Lin, Yong Liu, Jianping Chen, Fei Yan

**Affiliations:** 1 College of Plant Protection, Nanjing Agricultural University, Nanjing, 210095, PR China; 2 State Key Laboratory for Managing Biotic and Chemical Threats to the Quality and Safety of Agro-products, Institute of Plant Virology, Ningbo University, Ningbo, 315211, PR China; 3 Key Laboratory of Biotechnology in Plant Protection of MOA and Zhejiang Province, Zhejiang Academy of Agricultural Sciences, Hangzhou 310021, PR China; 4 Hunan Institute of Plant Protection, Changsha 410125, PR China

In the published version of this article the affiliation of Jinping Zhao was incorrectly given as State Key Laboratory for Managing Biotic and Chemical Threats to the Quality and Safety of Agro-products, Institute of Plant Virology, Ningbo University, Ningbo, 315211, PR China.

The correct affiliation for Jinping Zhao is Key Laboratory of Biotechnology in Plant Protection of MOA and Zhejiang Province, Zhejiang Academy of Agricultural Sciences, Hangzhou 310021, PR China.

This has been corrected in the author list and affiliations presented above and also in the published version of record.

The authors apologise for any inconvenience caused.

